# Efficacy and Safety of Neem Oil for the Topical Treatment of Bloodsucking Lice *Linognathus stenopsis* in Goats under Field Conditions

**DOI:** 10.3390/ani13152541

**Published:** 2023-08-07

**Authors:** Alessio Cotticelli, Roberta Matera, Nadia Piscopo, Antonio Bosco, Salvatore Claps, Paola Del Serrone, Aloma Zoratti, Elisa Castaldo, Vincenzo Veneziano, Domenico Rufrano, Gianluca Neglia, Francesco Buono

**Affiliations:** 1 Department of Veterinary Medicine and Animal Production, University of Naples Federico II, Via F. Delpino 1, 80137 Naples, Italy; alessio.cotticelli@unina.it (A.C.); roberta.matera@unina.it (R.M.); nadia.piscopo@unina.it (N.P.); antonio.bosco@unina.it (A.B.); vincenzo.veneziano@unina.it (V.V.); neglia@unina.it (G.N.); francesco.buono@unina.it (F.B.); 2 CREA Research Centre for Animal Production and Aquaculture, Bella Muro, 85051 Bella, Italy; salvatore.claps@crea.gov.it (S.C.); drufrano@tiscali.it (D.R.); 3 CREA Research Centre for Animal Production and Aquaculture, Via Salaria 31, Monterotondo, 00016 Rome, Italy; serrone85@gmail.com; 4 Department of Agricultural, Environmental and Animal Science, University of Udine, 33100 Udine, Italy; zoratti.aloma@spes.uniud.it

**Keywords:** goat, pediculosis, ectoparasites, *Linognathus stenopsis*, neem oil, bioactive plant compound

## Abstract

**Simple Summary:**

Goats are susceptible to ectoparasite infection, and lice control is based on studies conducted in cattle and sheep through the topical application of several synthetic insecticides such as organophosphates, carbamates, and pyrethroids. However, the overuse of these compounds has led to insect resistance phenomena. Thus, natural insecticides could be preferred, considering that synthetic insecticides and their metabolites negatively impact the environment and human and animal health.

**Abstract:**

The aim of the present study was to evaluate the efficacy and safety of neem oil on caprine pediculosis and on kids’ growth performances. The neem (*Azadirachta indica*) belongs to the Meliaceae family, and in Eastern countries it is mainly considered for the insecticidal activities of the kernel oil. The neem seeds contain bioactive principles, such as azadirachtin A, salannin, nimbin, and nimbolide. The trial was carried out on 24 kids, 120 days old, maintained in open yards. Animals were divided in 4 homogeneous groups (*n* = 6 animals/group) based on age, louse count, body condition score (BCS) and live body weight: Control Group (C, saline NaCl, 0.9%), Neem Group 1 (NO-100, 100 mL of neem oil per 10 kg), Neem Group 2 (NO-200, 200 mL/10 kg), Neem Group 3 (NO-300, 300 mL/10 kg). The treatments were performed by spraying the insecticide on the goat’s body. The study lasted 56 days, and weekly, the kids underwent louse count, BCS and body weight determination, and FAMACHA score. Data were analyzed by ANOVA for repeated measures. The species of lice identified was *Linognathus stenopsis*. Kids belonging to NO-200 and NO-300 showed a stronger reduction of louse count throughout the study (>95%). The daily weight gain recorded was significantly higher (*p* < 0.05) in NO-300 than C. No differences were found for BCS and FAMACHA scores. The results of this trial showed that the administration of neem oil to control caprine pediculosis caused by sucking lice represents an alternative to synthetic compounds.

## 1. Introduction

Europe holds only 1.9% of the world goat population, and the European goat sector is mainly linked to milk production and industrial cheese manufacturing, producing 15.1% and 35.1% of goat milk and goat cheese, recorded worldwide, respectively [1,2], representing an important economic source, especially in the countries of the Mediterranean basin [3]. In Europe, goats are raised mainly in Greece, Spain, Romania, and France (3.58, 2.65, 1.59, and 1.24 million heads, respectively), although more than 1 million heads are present also in Italy [4]. Furthermore, Europe can count on the widest caprine biodiversity (which represents the strength of this livestock subsector), with 187 goat breeds making up to 33% of the goat breeds recognized worldwide [5]; this is due to the ecosystem diversity.

Throughout the old continent, large dairy industries coexist with small local ones and artisanal farm dairies, transforming goat milk into cheese or yogurt. Many scientists focused on functional properties of the goat milk [6], including not only high nutritional value but also therapeutic potential and dietary characteristics [7]. Goat’s milk has great digestibility, buffer capacity, alkalinity, and therapeutic values (such as prebiotics and anticarcinogenic activities due to the high level of oligosaccharides and conjugated linoleic acid, respectively). This type of milk represents a nutraceutical health drink to be given to people who are intolerant to cow’s milk [7].

However, because of their breeding conditions, small ruminants are often infested by both endo and ectoparasites that can have a great negative impact on health status and animal productions [8]. The most important ectoparasites that affect the productivity of goats are lice and ticks. A small number of lice could be defined as a normal component of the skin fauna, however, heavy infestations can lead to severe symptom manifestations. As a matter of fact, caprine pediculosis represents a common disease in goats bred in an intensive grazing system [9]. Goats can be infested by bloodsucking lice (Anoplura, *Linognathus* spp.) and chewing or biting lice (Mallophaga, *Bovicola/Damalinia* spp.). Lice are more prevalent during winter and early spring, on animals left in colder climates, and in mountain and coastal regions [10].

Chewing and bloodsucking lice are obligate ectoparasites and survive only a few hours far away from their hosts and thus avoid dropping down from them [10]. Transmission from animal to animal occurs by direct physical contact, although it can also occasionally occur through flies [11]. *Linognathus*’ life cycle lasts 2–5 weeks, whereas *Bovicola*’s is completed in 3 weeks. Lice are responsible for vehiculating several microorganisms such as bacteria, although the main effect of the infestation is the direct damage to the host, causing scratching, rubbing, alopecia, papulo-crustous dermatitis, self-excoriation, anemia, and, as an indirect consequence, poor body condition score (BCS) and lower milk and meat productions [11,12].

Treatment of caprine pediculosis is extrapolated from studies conducted on cattle and sheep, and it is based on the employment of several synthetic insecticides used topically as a pour-on formulation, with the most important being pyrethroids and macrocyclic lactones [13,14,15]. However, the indiscriminate use of insecticides can harm human and animal health, increasing the risk of chemical residual in animals and animal-derived products [10].

In this context, alternatives to conventional synthetic insecticides may be natural products with pediculicidal properties developed from biologically active compounds of plant origin. Plant-based biopesticides exhibit various effects on insect populations, reducing their developmental, survival, and reproductive rate [16,17]. Among the plants that have insecticidal activity are *Azadirachta indica* A. Juss (Meliaceae), *Melia azedarach* L. (Meliaceae), *Lantana camara* L. (Verbenaceae), *Eucalyptus* spp. (Myrtoideae), *Solanum nigrum* L. (Solanaceae), *Origanum vulgare* L., and *Thymus vulgaris* L. (Lamiaceae) [18,19].

The neem (*Azadirachta indica*) is a tree belonging to the Meliaceae family native of Bangladesh, India, and Birmania. In Eastern countries, it is mainly considered for use because of the insecticidal activities of the kernel oil against different insect species [13,20]. This is due to the presence of more than 35 biologically active principles, of which azadirachtin A is the most important [21]. Azadirachtin shows antifeedant activity, blockage of morphogenetic peptide hormone release, and a direct detrimental effect on insect tissues [22,23]. Neem-based products rarely cause resistance phenomena due to their multiple modes of action against pests and the low toxicity rates [24,25,26].

In goats, antiseptic properties of the neem oil were evaluated on wounds characterized by bacterial infection, showing that the neem oil is effective and safe for the treatment of wounds [27]. However, few studies have been undertaken to assess the potential of neem products as insecticides for the control of ectoparasite infestations.

This study was conducted to evaluate (i) tolerance and safety of neem oil treatment; (ii) effective dosage in caprine pediculosis; and (iii) quantification of its impact on kids’ growth performances.

## 2. Materials and Methods

### 2.1. Study Farm

The study was performed between January and March 2019 in CRA-ZOE (Council for Agricultural Research and Analysis of Agricultural Economy Analysis) experimental farm located in Bella Muro, Potenza province (Basilicata region—southern Italy), which consisted of approximately 200 goats raised for milk production.

Goats were bred in the pasture during the day and returned to stalls in the afternoon (about from 7:00 a.m. to 4:00 p.m.). All animals were naturally infested by ectoparasites. Historically, lice control was performed individually two times/year (February and October) using pour-on application of synthetic pyrethroids (deltamethrin) (Butox 7.5 pour-on) and macrocyclic lactone (eprinomectin) (Eprinex pour-on 0.5%).

### 2.2. Farm Parasitological Status

For taxonomic identification, live lice (*n* = 50) were collected 1 week before (day −7) the beginning of the trial from 5 randomly selected animals. The identification has been performed as follows: ethanol fixation (70%), immersion in KOH (4%) for 7–8 h, ethanol (70%) and glycerol fixation, and mounted on a slide. The slides were examined under both a stereomicroscope (Leica S9i, Leica Microsystems GmbH, Tokyo, Japan) and an optical microscope (Leica DM 750, Leica Microsystems GmbH) to compare louse’s morphometrical data with the literature [28].

### 2.3. Study Animals

The trial was performed on 24 female Cashmere kids with a mean age of 120.88 ± 1.28 days. One week before the beginning of the study, each animal was weighed and fed with the same alimentary regimen: wheat sharps, wheat flour, maize, soybean meal, cane molasses, calcium carbonate, dehydrated beet pulp, soybean hulls, sodium bicarbonate, sodium chloride, dicalcium phosphate, and mixed meadow hay. Chemical characteristics of both concentrate and hay are reported in Table 1 and Table 2, respectively.

The concentrate amount for each study group was administered to meet energy and protein requirements of kids; therefore, it progressed from 0.4 kg/head at the beginning of the trial to 0.5 kg/head in the end. Hay and water were given ad libitum.

### 2.4. Louse Counting Procedures

Three days before (day −3) the first neem oil application (day 0), each animal was examined, and the individual louse count was performed at eight predilection body sites. The predilection body sites were determined based on louse distribution studies [29,30,31]. The predilection sites were as follows: right and left cheek (5 × 10 cm area), right and left ear (5 × 10 cm area), right and left neck and dewlap (10 × 10 cm area), right and left withers (10 × 10 cm area), right and left foreleg (axilla; 10 × 10 cm area), right and left back (10 × 10 cm area), right and left hind leg (inguen; 10 × 10 cm area), tailhead and perineum (10 × 10 cm area). In case no louse was found in any predilection site, the whole body was inspected. Counting was performed with the naked eye by the same operators throughout the study aided by a head-mounted, high-intensity focused light source and by a magnifying lens (10× magnification).

### 2.5. Groups and Treatment

According to body weight (BW), FAMACHA, BCS, and louse count, kids were divided into four homogenous groups (six animals/group): one control group (C group) and three experimental groups treated with a neem oil solution containing 0.35% azadiracthin A.

Goat kids belonging to the C group were treated with saline solution (NaCl 0.9%), goat kids belonging to Neem Group 1 (NO-100) were treated at the dosage of 100 mL/10 kg/BW, goat kids belonging to Neem Group 2 (NO-200) were treated at the dosage of 200 mL/10 kg/BW, and goat kids belonging to Neem Group 3 (NO-300) were treated at the dosage of 300 mL/10 kg/BW.

Neem oil chemical characteristics and metabolomic fingerprint were determined by high performance thin layer chromatography according to previously published methods [32,33].

Animals belonging to the C group were submitted to the same handlings of experimental ones. Both neem oil and saline solution treatments were carried out by nebulization. On day 0, the study animals received, in a single administration, the neem oil solution sprayed through plastic bottles with a finger pump spray on the head along the midline of the back from the withers to the tailhead.

Experimental groups were kept in distinct paddocks with an empty one (about 5 m) between them, to avoid contact and re-infestation, through the entire study period.

### 2.6. Treatment Efficacy

The study followed the procedures proposed by the World Association for the Advancement of Veterinary Parasitology (WAAVP) to evaluate the efficacy of ectoparasiticides in ruminants [29,31].

Individual louse counts were performed weekly (days 0, 7, 14, 21, 28, 35, 42, 49, and 56) by summing all predilection site counts for each goat kids for 8 weeks (to cover a total of 2 complete parasite life cycles).

The arithmetic mean of each goat kid’s cumulative louse count was calculated for each inspection, and neem oil biocide activity was evaluated weekly (7, 14, 21, 28, 35, 42, 49, and 56 days) in terms of percent louse reduction, calculated by the Abbott formula [34]:

Efficacy = [(mC − mT)/mC] × 100

where mC and mT are the mean numbers of lice on control and experimental animals, respectively. According to WAAVP guidelines, a percentage reduction > 90% was considered effective [31].

### 2.7. Tolerance and Safety Assay

Alongside biocide activity, dermatological conditions were evaluated using the standard Draize skin irritation scoring system [35]. Each animal received a score from 0 to 3 based on skin conditions: 0—no alterations; 1—erythema/abrasion; 2—oedema; 3—hair loss on application site. To evaluate potential lesions due to neem oil, animals were kept under observation for 4 h after treatment and then once a week until the end of the trial.

### 2.8. Productive Performances

BW and BCS were measured before the beginning of the study (day −3) and then weekly (days 0, 7, 14, 21, 28, 35, 42, 49, and 56) until the end of the study period. BCS was determining using a five-point scale (1–5) with 0.5 increments [36]. Data concerning goat kids’ weight were recorded, and the daily weight gain (DWG) was calculated accordingly. Individual feedstuff and refusals were sampled once per week and analyzed according to the Association of Official Analytical Chemists (AOAC) [37]. Energy values (milk forage units = 1700 kcal) were calculated using equations provided by the INRA [38].

### 2.9. FAMACHA

FAMACHA score evaluation (acronymous of Dr. Francois “Faffa” Malan (FAffa MAlan CHArt) [39] was performed simultaneously to louse counts: 3 days before the beginning of the study (day −3) and then weekly (days 0, 7, 14, 21, 28, 35, 42, 49, and 56). It consists of a laminated chart with five color categories showing worsening degrees of anemia (score from 1 to 5); these colors are compared to the conjunctivae of goats under examination [40].

### 2.10. Statistical Analyses

Statistical analysis was performed using SPSS (28.0) for Windows 10.0 (SPSS Inc., Chicago, IL, USA). Data on louse count, BCS, weight, FAMACHA, and DWG were analyzed by analyses of variance (ANOVA) with treatment as the main factor, kids were the random factors, and the day was the repeated measure. The single time points were also compared using paired samples *t*-tests. *p* values < 0.05 were considered to indicate significant differences between means.

## 3. Results

### 3.1. Parasitological Results

Morphological features of louse allowed the identification of *Linognathus stenopsis* as responsible for infestation in all study animals. Louse count at the beginning of the trial (day 0) showed no differences between the groups (*p* = 0.87). The infestations were highly variable between the study goat kids on day 0, with per animal counts ranging from 18 to 318 lice.

The following table (Table 3) reports the arithmetic mean louse counts performed on the goat kids belonging to the four experimental groups and the corresponding percentages of reduction at each time study point.

**Figure 1:**
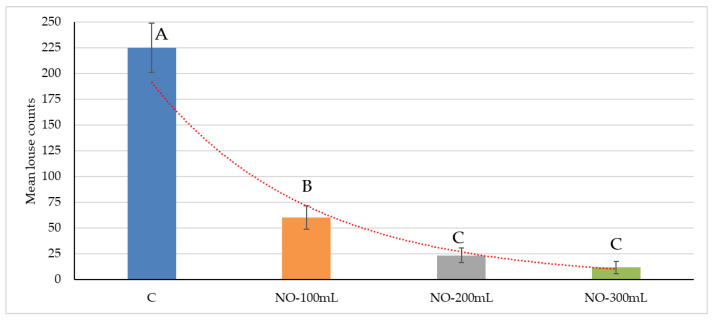
Louse counts between the four experimental groups throughout the study. C: control group; NO-100: kids treated at the dosage of 100 mL/10 kg/BW; NO-200: kids treated at the dosage of 200 mL/10 kg/BW; NO-300: kids treated at the dosage of 300 mL/10 kg/BW. Data are presented as mean ± SE (^A,B,C^ *p* < 0.01).

The mean louse count ± standard error (SE) throughout the study was 224.65 ± 23.95 (C group), 59.93 ± 11.43 (NO-100 group), 23.24 ± 7.09 (NO-200 group), and 11.52 ± 6.05 (NO-300 group). Louse counts showed differences between NO-100 and NO-200 as well as NO-100 and NO-300 (*p* < 0.01); and between the three treatments and the control group (Figure 1). Moreover, although there were no significant differences between the NO-200 and NO-300 groups, the application of neem oil at the dosage of 300 mL/10 kg/BW showed greater efficacy than the 100 mL/10 kg/BW and 200 mL/10 kg/BW.

The dermal safety study on the neem oil showed the absence of adverse effects caused by the treatment (Draize score = 0). No abnormal general health conditions related to all treatments in the three study groups were observed during the study.

### 3.2. Productive Performance

Body weight (kg), BCS, and FAMACHA score were in the physiological range for goat kids, and no significative differences were highlighted between the four study groups at the beginning of the trial (day 0).

Mean body weight values were 9.32 ± 1.01 (C group), 9.12 ± 1.30 (NO-100 group), 9.10 ± 1.19 (NO-200 group), and 9.20 ± 1.29 (NO-300 group).

Mean BCS ± SE were 3.33 ± 0.33 (C group), 3.33 ± 0.31 (NO-100 group), 3.42 ± 0.35 (NO-200 group), and 3.42 ± 0.49 (NO-300 group).

**Figure 2:**
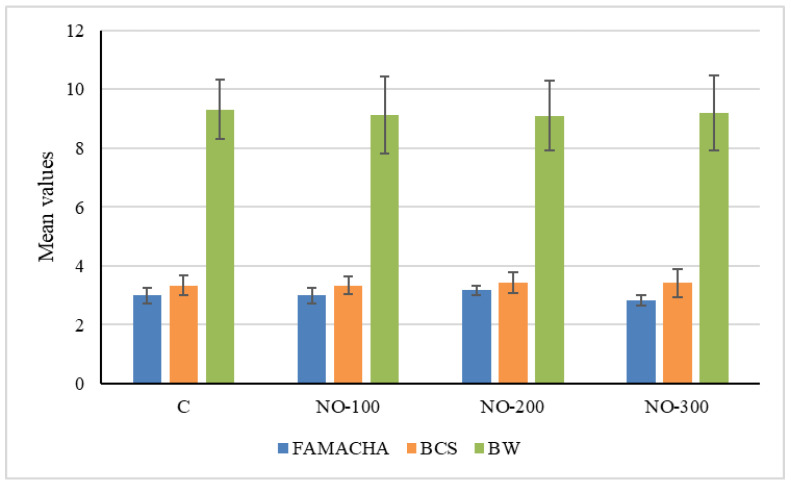
FAMACHA, BCS, and BW (kg) of the four groups at the beginning of the trial (day 0). C: control group; NO-100: kids treated at the dosage of 100 mL/10 kg/BW; NO-200: kids treated at the dosage of 200 mL/10 kg/BW; NO-300: kids treated at the dosage of 300 mL/10 kg/BW. All data are presented as mean ± SE.

Mean FAMACHA scores ± SE were 3.00 ± 0.26 (C group), 3.00 ± 0.26 (NO-100 group), 3.17 ± 0.17 (NO-200 group), and 2.83 ± 0.17 (NO-200 group) (Figure 2).

During the study (day 0–day 56), the mean BWs ± SE were 10.80 ± 0.32 (C group), 11.82 ± 0.52 (NO-100 group), 11.49 ± 0.51 (NO-200 group), and 12.67 ± 0.52 (NO-300 group).

Similarly, the mean DWGs (g/die) were 57.44 ± 19.73 (C group), 87.5 ± 17.04 (NO-100 group), 89.29 ± 13.99 (NO-200 group), and 107.74 ± 17.32 (NO-300 group). The goat kids belonging to the NO-300 group showed a weight (*p* < 0.01) and a daily weight gain (*p* < 0.05) higher than the C group.

**Figure 3:**
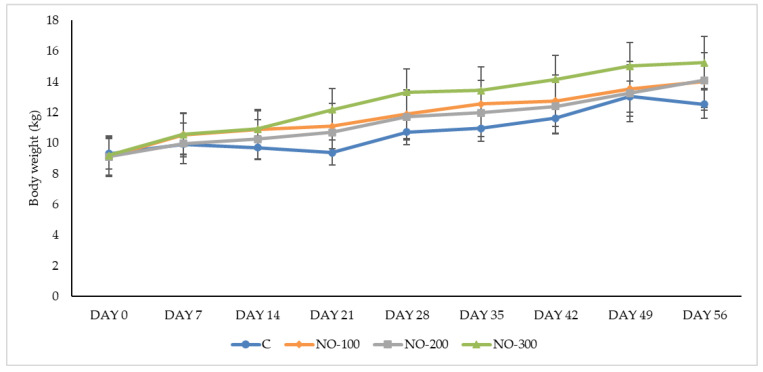
Body weights (kg) recorded throughout the trial for study groups. C: control group; NO-100: kids treated at the dosage of 100 mL/10 kg/BW; NO-200: kids treated at the dosage of 200 mL/10 kg/BW; NO-300: kids treated at the dosage of 300 mL/10 kg/BW. Data are presented as means ± SE.

Conversely, no differences were recorded between the weekly body weights (kg) of the animals belonging to the four study groups (Figure 3).

Weekly weight gains (Figure S1) differed significantly in the third week of the study between the NO-300 and C groups (*p* < 0.01) and in the last week between the NO-200 and the other three groups.

**Figure 4:**
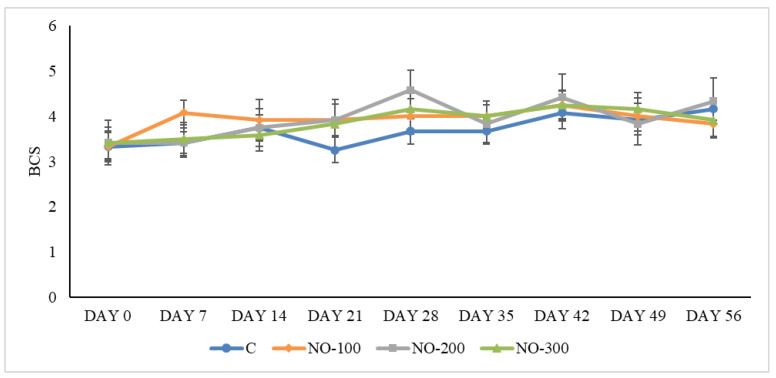
BCS from day 0 to 56 for the four study groups. C: control group; NO-100: kids treated at the dosage of 100 mL/10 kg/BW; NO-200: kids treated at the dosage of 200 mL/10 kg/BW; NO-300: kids treated at the dosage of 300 mL/10 kg/BW. Data are presented as means ± SE.

BCS values were similar between groups during the study (day 0–day 56) and always remained in the physiological range of the species: 3.69 ± 0.28 (C group), 3.93 ± 0.35 (NO-100 group), 3.94 ± 0.42 (NO-200 group), and 3.87 ± 0.40 (NO-300 group) (Figure 4).

No statistical differences were recorded between BCS values of the animals belonging to the four study groups.

**Figure 5:**
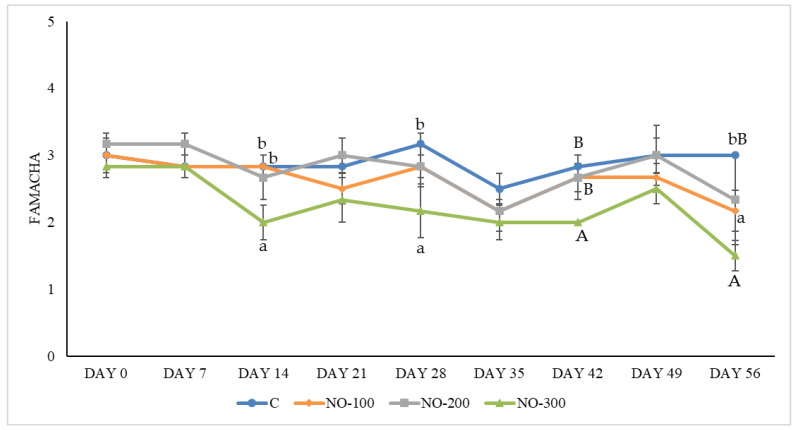
FAMACHA score for the study groups from day 0–day 56. C: control group; NO-100: kids treated at the dosage of 100 mL/10 kg/BW; NO-200: kids treated at the dosage of 200 mL/10 kg/BW; NO-300: kids treated at the dosage of 300 mL/10 kg/BW. Data are presented as means ± SE (^A,B^ *p* < 0.01; ^a,b^ *p* < 0.05).

The mean FAMACHA scores during the study (day 0–day 56) were 2.89 ± 0.06 (C group), 2.63 ± 0.08 (NO-100 group), 2.78 ± 0.12 (NO-200 group), and 2.24 ± 0.09 (NO-300 group). However, the NO-300 group showed the lowest FAMACHA score throughout the trial, and significant differences emerged at days 14, 28, 42, and 56 (Figure 5).

## 4. Discussion

To the best of the authors’ knowledge, our study is one of the first to evaluate the efficacy of neem oil against caprine pediculosis caused by *Linognathus stenopsis*.

Ectoparasitism is a common and often underestimate issue in goat farming [9]. Considerable deterioration in growth and production performance of animals can be correlated to ectoparasites, resulting in reduced profits from livestock rearing [41]. Both direct (reduced quality of goat products, nutritional and metabolic containments, and lowered production performance) and indirect effects of lice infestations (immune regulations and oxidative impairments) [14] point out a detrimental effect of pediculosis on goat welfare, which ends up impairing the fulfilment of their ethological requirements.

The results of the present study showed that the administration of neem oil is effective for controlling *Linognathus stenopsis* infestations in goats. The neem oil administered at a dose of 300 mL/10 kg/BW showed a higher efficacy in terms of louse count reduction, suggesting a dose-dependent manner of the complex. In fact, NO-100 mL did not reach such a threshold (69.1% at day 49 until a maximum of 89.0% at day 14 and a mean value of 83.4%). The administration of neem oil at dosage of 200 mL/10 kg/BW was effective from day 7 until day 56 (louse count reduction always higher than 90%, with a mean of 96.99%). Finally, NO-300 showed a peak of reduction (100%) between day 28 and 42, keeping an average efficiency of 99.9%. Differences concerning louse counts proved the efficiency of neem oil treatment at doses of 200 and 300 mL/10 kg/BW: 18.5 and 28.7 vs. 72.1 for NO-300, NO-200, and NO-100, respectively (*p* < 0.01).

Our results are similar to those obtained in Angora goats naturally infected with chewing louse *Damalinia limbate*, in which a solution of Neem Azal^®^ determined a reduction in louse density of 76–96% from 2 to 18 weeks after treatment [17].

In New Zealand, the administration of neem seed extract (50% azadirachtin) (neem oil diluted with water—2 g/animal) was effective for controlling *Bovicola* (*Damalinia*) *ovis* in sheep [42]. Moreover, a study performed in sheep in Australia showed that a single administration of neem seed extract at different concentrations reduced infestation by 98–100% and prevent the reinfestation of *Damalinia ovis* for 6 months [43]. In horses heavily infected by *Werneckiella equi,* the administration of neem seed extract was effective in controlling lice density [44]. Moreover, in dogs, two neem seed preparations, one for large dogs (concentration of 1:33 with tap water) and one for small dogs (10% neem seed extract and 90% shampoo) were effective for controlling the chewing lice *Trichodectes canis* [45].

In goats, neem seed extract (10% water solution) reduced tick infestation [46]. In beef heifers, pour-on administration of neem oil was ineffective for reducing the mean infestation by *Rhipicephalus* (*Boophilus*) *microplus* [47], whereas it showed an efficiency ranging from 60 to 75% against *R. microplus*, *Hyalomma anatolicum anatolicum,* and *R. haemaphysaloides* in bovines and buffaloes [48].

Concerning productive performances, the C group had a DWG of 57.44 ± 19.73 g/head/die during the trial; no significant differences emerged between the three experimental groups (87.50 ± 17.04, 89.29 ± 13.99, and 107.74 ± 17.32 g/head/die, respectively, for NO-100, NO-200, and NO-300). On the other hand, the average DWG of NO-300 was significantly higher than the C one (*p* < 0.05), suggesting a direct correlation between louse density and growth performance of goat kids. Moreover, the average weight value throughout the study confirms such data, highlighting a difference (*p* < 0.01) between the C and NO-300 groups (10.80 ± 0.32 and 12.67 ± 0.52, respectively). The correlation between growing performances (in terms of both weight and DWG) and louse density is in agreement with previous studies [14] and underlines the need to meet ethological and welfare requirements to avoid loss of production performance of goats. These data differ from those reported in beef heifers, in which animals treated with homeopathic preparations (including neem oil) have not gained weight compared to positive control animals (treated with 10% moxidectin and with an acaricidal formulation of cypermethrin) [47].

BCS was in the physiological range for goat kids during the study period, indicating that louse infestations do not represent a risk factor, as also reported by Seyoum et al. [49].

The results of this study prove the pediculicidal potential of neem oil and its feasibility against *L. stenopsis* infestations in goats. As a matter of fact, combining parasite density reduction and louse count findings throughout the trial, it is possible to infer a dose-dependent effect of insecticides. Indeed, the Abbott formula revealed a full efficacy (days 28, 35, and 42) only for the dosage of 300 mL/10 kg/BW, whereas lower doses depicted a decrement in louse reduction.

## 5. Conclusions

The results of this study showed that the administration of neem oil (300 mL/10 kg/BW) is useful to control caprine pediculosis caused by sucking lice (*L. stenopsis*). Neem oil is confirmed to be a trustworthy approach against ectoparasite infestations, reducing parasite count and improving goat kids’ growth performances.

Further studies should be focused on the toxicity, pharmacokinetic parameters, and dose–response relationship of neem oil, possibly involving a larger sample size.

## Figures and Tables

**Table 1:** Chemical characteristics of concentrate for feeding the kids.

Composition	%
Dry matter (DM)	87.5
Crude protein (CP)	16.5
Ether extract (EE)	3.5
Neutral detergent fiber (NDF)	26.4
Acid detergent fiber (ADF)	14.5
Acid detergent lignin (ADL)	6.7
Crude fiber (CF)	6.5
Ash	8.5

**Table 2:** Chemical characteristics of hay for feeding the kids.

Composition	%
Dry matter (DM)	88.7
Crude protein (CP)	7.4
Ether extract (EE)	1.2
Neutral detergent fiber (NDF)	59.6
Acid detergent fiber (ADF)	39.9
Acid detergent lignin (ADL)	7.7
Crude fiber (CF)	31.9
Ash	5.9

**Table 3:** Louse counts (arithmetic mean ± standard error) and efficacy (percent louse reduction) for the goat kids infected by *Linognathus stenopsis* at each time study point.

	C	NO-100		NO-200		NO-300	
Time	Lice Count	Lice Count	Efficacy (%)	Lice Count	Efficacy (%)	Lice Count	Efficacy (%)
Day 0	97.83 ± 31.95	124.33 ± 46.86	-	138.00 ± 41.11	-	101.50 ± 40.89	-
Day 7	230.83 ± 75.89	34.83 ± 19.72	84.9	9.00 ± 2.02	96.1	0.50 ± 0.50	99.8
Day 14	233.17 ± 70.49	31.00 ± 15.45	86.7	6.67 ± 1.82	97.1	0.67 ± 0.49	99.7
Day 21	303.17 ± 133.35	43.17 ± 23.50	85.8	3.67 ± 1.28	98.8	0.17 ± 0.17	99.9
Day 28	233.50 ± 58.40	35.83 ± 20.56	84.7	3.50 ± 1.18	98.5	0.00 ± 0.00	100
Day 35	264.00 ± 70.92	54.83 ± 34.71	79.2	5.00 ± 1.71	98.1	0.00 ± 0.00	100
Day 42	173.17 ± 53.66	42.17 ± 21.75	75.6	10.83 ± 5.39	93.7	0.00 ± 0.00	100
Day 49	242.83 ± 77.01	100.00 ± 60.87	58.8	14.33 ± 4.51	94.1	0.33 ± 0.21	99.9
Day 56	243.33 ± 49.67	73.17 ± 39.62	69.9	18.17 ± 6.07	92.5	0.50 ± 0.50	99.8

C: control group; NO-100: kids treated at the dosage of 100 mL/10 kg/BW; NO-200: kids treated at the dosage of 200 mL/10 kg/BW; NO-300: kids treated at the dosage of 300 mL/10 kg/BW.

## Data Availability

The data presented in this study are available on request from the corresponding author.

## Supplementary Materials

The following supporting information can be downloaded at: https://www.mdpi.com/article/10.3390/ani13152541/s1, Figure S1: Weekly weight gain (g/die) during the trial for the four study groups (C, NO-100 mL, NO-200 mL, NO-300 mL). Different letters differ significantly (^A,B^ *p* < 0.01; ^a,c^ *p* < 0.05). Data are presented as means ± SE.
