# Supplementary figures and images for: Efficacy and Safety of Neem Oil for the Topical Treatment of Bloodsucking Lice Linognathus stenopsis in Goats under Field Conditions
Source: Animals (Basel). 2023 Aug 7;13(15):2541. doi: 10.3390/ani13152541 (PMC10417451; doi:10.3390/ani13152541)

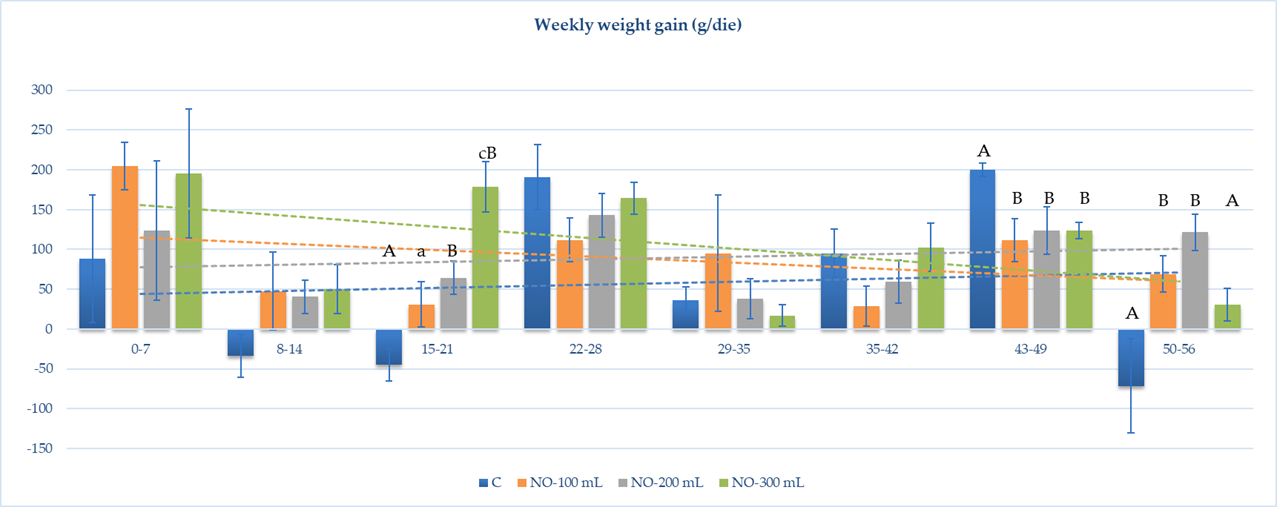

Supplement: Supplementary file 1 [file animals-13-02541-s001.zip › animals-2539119-supplementary.tif]
